# Sildenafil affects the human Kir2.1 and Kir2.2 channels at clinically relevant concentrations: Inhibition potentiated by low Ba^2+^

**DOI:** 10.3389/fphar.2023.1136272

**Published:** 2023-02-02

**Authors:** Akimasa Iijima, Olga Švecová, Jan Hošek, Roman Kula, Markéta Bébarová

**Affiliations:** ^1^ Department of Physiology, Faculty of Medicine, Masaryk University, Brno, Czech Republic; ^2^ Department of Molecular Pharmacy, Faculty of Pharmacy, Masaryk University, Brno, Czech Republic

**Keywords:** sildenafil, arrhythmia, barium, inward rectifier, Kir2.1, Kir2.2

## Abstract

Sildenafil (Viagra), the first approved and widely used oral drug for the treatment of erectile dysfunction, was occasionally associated with life-threatening ventricular arrhythmias in patients. Since inward rectifier potassium current (*I*_K1_) may considerably contribute to this arrhythmogenesis, we investigated the effect of sildenafil on the human Kir2.1 and Kir2.2, the prevailing subunits forming the ventricular *I*_K1_ channels. Experiments were performed by the whole-cell patch clamp technique at 37°C using Chinese hamster ovary cells transiently expressing the human Kir2.1 and Kir2.2 channels. Changes of both the inward and outward current components (at −110 and −50 mV, respectively) were tested to be able to consider the physiological relevance of the sildenafil effect (changes at −110 and −50 mV did not significantly differ, results at −50 mV are listed below). A significant Kir2.1 inhibition was observed at all applied sildenafil concentrations (16.1% ± 3.7%, 20.0% ± 2.6%, and 15.0% ± 3.0% at 0.1, 1, and 10 μM, respectively). The inhibitory effect of 0.1 μM sildenafil was potentiated by the presence of a low concentration of Ba^2+^ (0.1 μM) which induced only a slight Kir2.1 inhibition by 5.95% ± 0.75% alone (the combined effect was 35.5% ± 3.4%). The subtherapeutic and therapeutic sildenafil concentrations (0.1 and 1 μM) caused a dual effect on Kir2.2 channels whereas a significant Kir2.2 activation was observed at the supratherapeutic sildenafil concentration (10 μM: 34.1% ± 5.6%). All effects were fully reversible. This is the first study demonstrating that sildenafil at clinically relevant concentrations inhibits both the inward and outward current components of the main human ventricular *I*_K1_ subunit Kir2.1. This inhibitory effect was significantly potentiated by a low concentration of environmental contaminant Ba^2+^ in agreement with recently reported data on rat ventricular *I*_K1_ which additionally showed a significant repolarization delay. Considering the similar subunit composition of the human and rat ventricular *I*_K1_ channels, the observed effects might contribute to sildenafil-associated arrhythmogenesis in clinical practice.

## Introduction

Sildenafil (Viagra), an inhibitor of phosphodiesterase type 5 (Turko et al., 1999), is the first approved oral drug for the treatment of erectile dysfunction. After its introduction to the market in 1998, millions of prescriptions for sildenafil have been written annually all over the world (Goldstein et al., 2019). Highlighted by the high prevalence of erectile dysfunction globally (Shaeer et al., 2017; Irfan et al., 2020) sildenafil has an enormous positive impact on men’s overall health. However, there are also concerns about the safety of this drug. Several cases of death following ventricular arrhythmias were reported among the patients treated with sildenafil (Rasmussen et al., 2007). Post-marketing surveillance analysis by US FDA reported deaths from severe cardiovascular events (myocardial infarction, arrhythmia, cardiac arrest, and collapse) and its temporal coupling to the use of sildenafil. In most of these patients, the sildenafil dose was standard, the age was below 65 years old, and no identifiable cardiac risk factor was reported (*e.g.* Azarbal et al., 2000). According to the November 1998 report, 44 of 77 cardiovascular deaths (where the time from drug ingestion to death was known) were precipitated within 4–5 h of the sildenafil use (Cheitlin et al., 1999; Zusman et al., 1999; Azarbal et al., 2000; Kloner 2000; Shinlapawittayatorn et al., 2005). To date, underlying mechanisms of ventricular arrhythmias associated with sildenafil have remained to be a matter of speculation.

The cardiac arrest that has been identified as one of the causes of sudden deaths described after the use of sildenafil (see above) is very likely related to the occurrence of ventricular fibrillation. Sildenafil was also demonstrated to decrease the ventricular fibrillation threshold in pigs (Kanlop et al., 2008). As well known, modification of the cardiac inward rectifier potassium (Kir) channels may considerably contribute to the genesis of this life-threatening arrhythmia (*e.g.,* Dhamoon and Jalife 2005; Piao et al., 2007; Jalife 2009; Sekar et al., 2009). For a recent review on Kir channels, see Reilly and Eckhardt (2021). It is important to note that the Kir channels play a key role in the restoration and stabilization of the resting membrane potential (at the end of phase three and during phase four of the cardiac action potential) and can be divided into various channel subtypes. Under basic physiological conditions, the most important one is the *I*_K1_ channel. As demonstrated in canine cardiomyocytes, *I*_K1_ did not significantly differ among individual layers of the ventricular myocardium (Li et al., 2002; Xiao et al., 2006) and was significantly higher in ventricular myocytes in comparison to atrial and Purkinje cardiac cells (Cordeiro et al., 2015). *I*_K1_ channel is preferentially formed by Kir2.1 and Kir2.2 subunits in the ventricle (Wang et al., 1998; Melnyk et al., 2002; Gaborit et al., 2007; Panama et al., 2007).

The effect of sildenafil on the Kir channel *I*_K1_ has been recently reported in freshly enzymatically isolated rat ventricular myocytes by our group (Macháček et al., 2022). Sildenafil caused a significant and reversible inhibition of both inward and outward components of the rat ventricular *I*_K1_ even at therapeutic concentrations. Moreover, the slight, but significant inhibitory effect of the subtherapeutic sildenafil concentration of 0.1 µM was substantially potentiated by a low concentration of Ba^2+^ (0.1 µM). Ba^2+^ is a potent *I*_K1_ inhibitor (at high µM concentrations) and environmental contaminant commonly detected in the plasma of healthy individuals (Łukasik-Głębocka et al., 2014). The combined effect of sildenafil and Ba^2+^ (both at 0.1 µM) resulted in a significant delay of action potential repolarization. To our knowledge, no data on the effect of sildenafil on *I*_K1_ in human cardiomyocytes are available.

This is the first study investigating changes in the expressed human Kir2.1 and Kir2.2 channels, the prevailing ventricular *I*_K1_ subunits, under the effect of sildenafil. Sildenafil was applied both alone (at concentrations between 0.1 and 10 µM) and in combination with Ba^2+^ at a concentration within the range identified in healthy individuals (0.1 µM). To better consider the physiological relevance of the sildenafil effect, changes of the currents at both −110 mV (the inward component) and −50 mV (the outward component) were tested.

## Materials and methods

### Cell culture and transfection

Chinese hamster ovary (CHO) cells were cultured at 37°C with 5% CO_2_ in Ham’s F-12 medium supplemented with 10% fetal calf serum and 0.005% gentamycin. The transfection of plasmids (pcDNA3-Hs_Kir2.1 and pcDNA3-Hs_Kir2.2 coexpressed with pIRES2_EGFP) was performed by TransFast Transfection Reagent (Sigma-Aldrich) approximately 48 h prior to measurements. The plasmids were provided as kind gifts by Dr. Marcel van der Heyden (Utrecht University, Netherlands; pcDNA3-Hs_Kir2.1 and pcDNA3-Hs_Kir2.2) and prof. Paul G.A. Volders (Maastricht University, Netherlands; pIRES2_EGFP).

### Solutions and chemicals

The Tyrode solution of the following composition (in mM) was used during measurements: NaCl 135, KCl 5.4, MgCl_2_ 0.9, HEPES 10, NaH_2_PO_4_ 0.33, CaCl_2_ 0.9, glucose 10 (pH was adjusted to 7.4 with NaOH). The solution was supplemented by specific inhibitors of several ionic currents to be the same one used during analogical measurements in rat cardiomyocytes (2 mM CoCl_2_, 50 mM tetraethylammonium chloride, 1 μM atropine, and 10 μM glibenclamide to inhibit calcium, delayed rectifier potassium, acetylcholine-sensitive potassium, and ATP-sensitive potassium currents, respectively; for details, see Macháček et al., 2022).

Sildenafil and BaCl_2_ were added to the Tyrode’s solution to obtain the final concentrations, 0.1, 1, and 10 μM in the case of sildenafil and 0.1 and 100 μM in the case of Ba^2+^.

The filling solution for the patch electrode contained (in mM): L-aspartic acid 130, KCl 25, MgCl_2_ 1, K_2_ATP 5, EGTA 1, HEPES 5, GTP 0.1, Na_2_-phosphocreatine 3 (pH 7.25 adjusted with KOH).

CoCl_2_ and atropine were prepared as 1 M and 1 mM stock solutions, respectively, in deionized water. Glybenclamide was prepared as 100 mM stock solution in DMSO (DMSO below 0.01% in both control and test solution). To prepare the TEA-containing stock solution, NaCl in the used Tyrode solution (described above) was replaced equimolarly by TEA.

The stock solutions of sildenafil (10 mM; DMSO as solvent) and BaCl_2_ (10 mM; deionized water as solvent) were added to the Tyrode solution to obtain working concentrations between 0.1 and 10 μM. For the simultaneous application of both drugs, 0.1 μM sildenafil and 0.1 μM barium were used. The concentration of DMSO in the final solution was kept below 0.01% in all experiments, thus, it should not exert any effect on the measured Kir currents by itself (Ogura et al., 1995; Bosch et al., 1999).

The solutions were applied in the vicinity of the measured cell *via* a gravity-operated perfusion system; the time to change the solution was approximately 2 s.

### Electrophysiological measurements and evaluation

**FIGURE 1:**
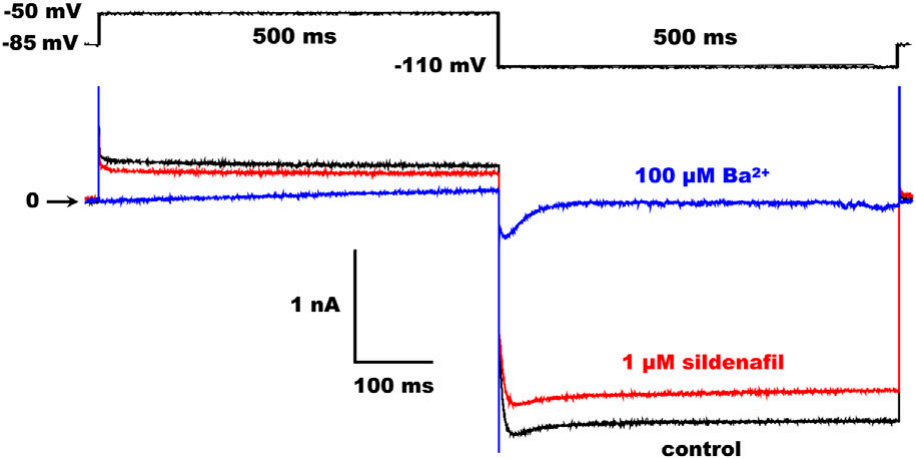
Scheme of the experimental protocol (upper panel) and representative Kir2.1 traces in control and under the effect of 1 µM sildenafil or 100 µM Ba^2+^ (lower panel).

CHO cells with GFP fluorescence were used for the current recordings applying the whole-cell patch-clamp technique in the voltage-clamp mode 24 h after the transfection. The patch pipettes were pulled from borosilicate glass capillary tubes and heat polished on a programmable horizontal puller (Zeitz-Instrumente, Germany). The resistance of the filled glass electrodes was below 2.5 MΩ to keep the access resistance as low as possible. The Axopatch 200 B equipment and pCLAMP 9.2 software (Molecular Devices) were used for the generation of experimental protocols and data acquisition. The series resistance was compensated up to 75%. The measured ionic currents were digitally sampled at 10 kHz and stored on the hard disc. Experiments were performed at 37 °C. The holding potential was −85 mV, and the stimulation frequency was 0.2 Hz in all experiments. Kir2.1 and Kir2.2 currents were evaluated as the current sensitive to 100 µM Ba^2+^ at the end of a 500-ms pulse, either to −110 mV or to −50 mV to check the inward or outward current component, respectively (for the experimental protocol and representative Kir2.1 traces in control conditions and under the effect of 1 µM sildenafil or 100 µM Ba^2+^, see Figure 1).

The average cell membrane capacitance *C*_m_ and access resistance *R*_a_ did not differ in CHO cells expressing the human Kir2.1 and Kir2.2 channels (Kir2.1: *C*_m_ = 15.7 pF, IQR 11.9–18.8, and *R*_a_ = 3.85 MΩ, IQR 2.81–6.00; Kir2.2: *C*_m_ = 15.8 pF, IQR 14.0–22.4, and *R*_a_ = 3.26 MΩ, IQR 2.50–5.21; *n* = 40 and 37, respectively, *p* > 0.05 in both cases; not illustrated). Since no significant correlation between the cell membrane capacitance and current magnitude was observed in both Kir2.1 and Kir2.2 (not illustrated), the current magnitude was not normalized by recalculation into the current density as previously recommended in such cases (Kula et al., 2020).

### Statistical analysis

In most data, normal data distribution was proved (Kolmogorov-Smirnov test) and the results are presented as the arithmetic mean ± S.E.M. from *n* cells (Origin, version 2022b, Origin Lab Corporation). Despite being normally distributed, non-parametric statistical tests were used to consider statistically significant differences among parameters, either because of comparison of data normalized to control or because the *F* value during ANOVA testing was not significant and, thus, *post hoc* tests were not eligible as recommended by Curtis et al. (2018); the Kruskal-Wallis test with the Dunn´s *post hoc* test in all shown graphs except for the graphs in Figure 4B and Figure 5B, right panels, where the Wilcoxon test was used). If the normal distribution was rejected (namely in *C*_m_ and *R*_a_), median and interquartile range (IQR) are listed and the non-parametric Mann-Whitney test was used to consider statistical differences between the parameter in the measured cells expressing Kir2.1 and Kir2.2. The curve fitting and statistical testing were performed using the GraphPad Prism, version 9.4.1 (GraphPad Software, Inc.); *p* < 0.05 was considered statistically significant.

## Results

The sildenafil-induced changes of Kir2.1 and Kir2.2 were studied both at −50 mV (*i.e.,* on the outward component which is small, but highly physiologically relevant) and at −110 mV (*i.e.,* on the inward component which is bigger, thus, measurement of its changes is more precise). No significant differences in the sildenafil effect were observed between both tested voltages.

### Significant inhibition of the human Kir2.1 channels under clinically relevant concentrations of sildenafil

**FIGURE 2:**
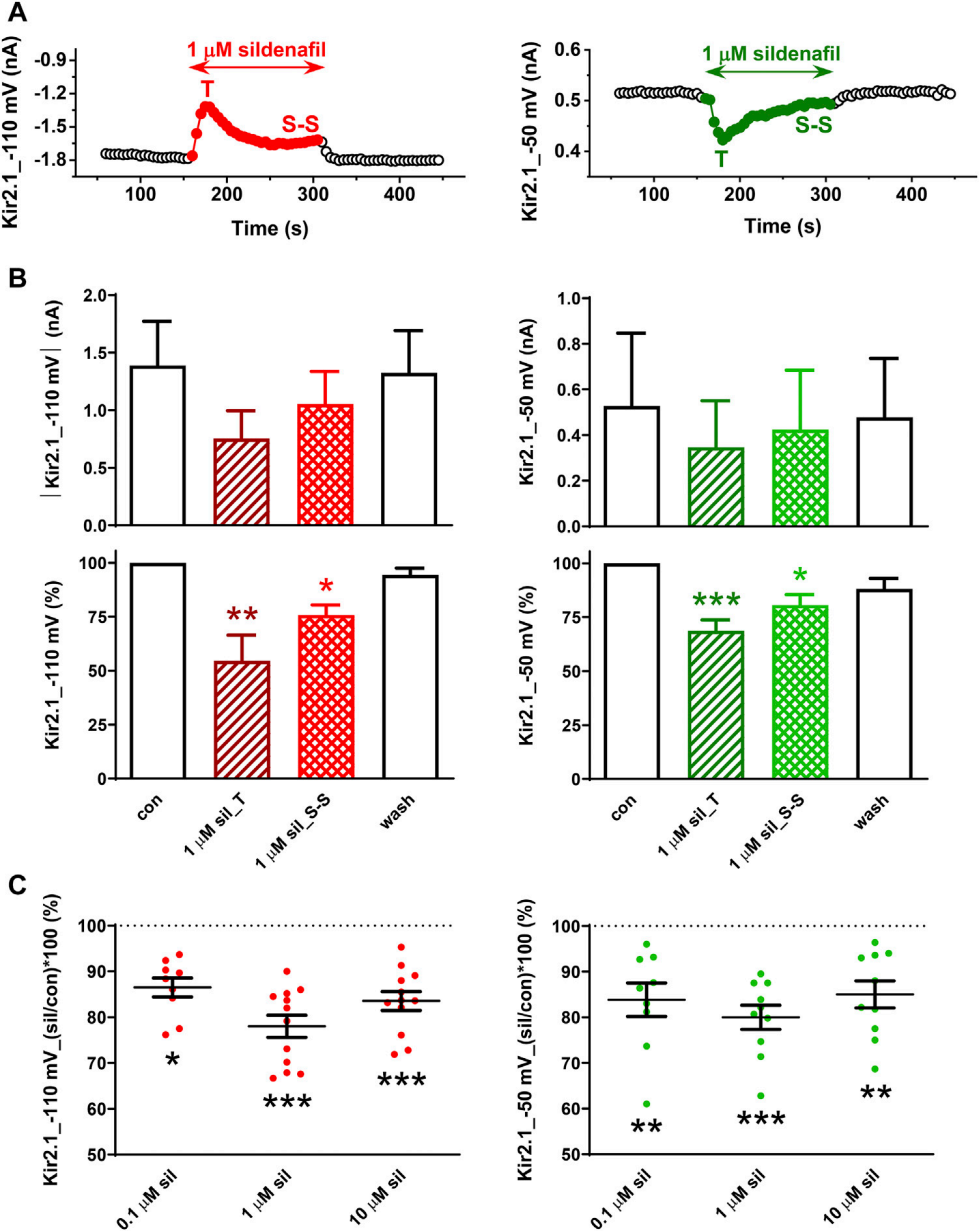
Effect of sildenafil on the human Kir2.1 channels. **(A)** Representative time courses of Kir2.1 changes in control (con), during application of 1 µM sildenafil (sil), and during the subsequent wash-out (wash); T—transient effect, S-S—steady-state effect. **(B)** Kir2.1 current and its average changes under 1 µM sildenafil (*n* = 6). **(C)** Concentration dependence of the steady-state effect of sildenafil on the human Kir2.1 (*n* = 9–12); *, **, and ***—statistical significance at *p* < 0.05, 0.01, and 0.001, respectively.

First, we tested the effect of 0.1–10 µM sildenafil on the human Kir2.1 channels. As illustrated in Figures 2A, B, application of sildenafil at the therapeutic concentration of 1 µM resulted in Kir2.1 inhibition that reached in half of the measured cells a transient peak (T) within 20.2 ± 3.0 s and 22.7 ± 3.1 s at both −110 and −50 mV (*n* = 6, *p* > 0.05), respectively, and subsequently decreased to the steady-state inhibition (S-S) in all cells. These effects were not significant when evaluated in the absolute values of the current magnitude (Figure 2B, upper panels) due to the high variability of the parameter, but they were significant when evaluated in the relative scale (Figure 2B, lower panels). The steady-state inhibition at 1 µM sildenafil reached 22.0% ± 2.4% and 20.0% ± 2.6% at −110 and −50 mV, respectively (Figure 2C).

A slightly lower, but still significant Kir2.1 inhibition was apparent at the other two tested concentrations, the subclinical concentration of 0.1 µM and the supratherapeutic concentration of 10 µM (13.5% ± 2.1% and 15.6% ± 2.2% steady-state inhibition at −110 mV, respectively, and 16.1% ± 3.7% and 15.0% ± 3.0% steady-state inhibition at −50 mV, respectively; Figure 2C). The effect at all concentrations did not differ at both tested voltages and was fully reversible during the subsequent wash-out.

### Dual sildenafil-induced changes in human Kir2.2 channels

**FIGURE 3:**
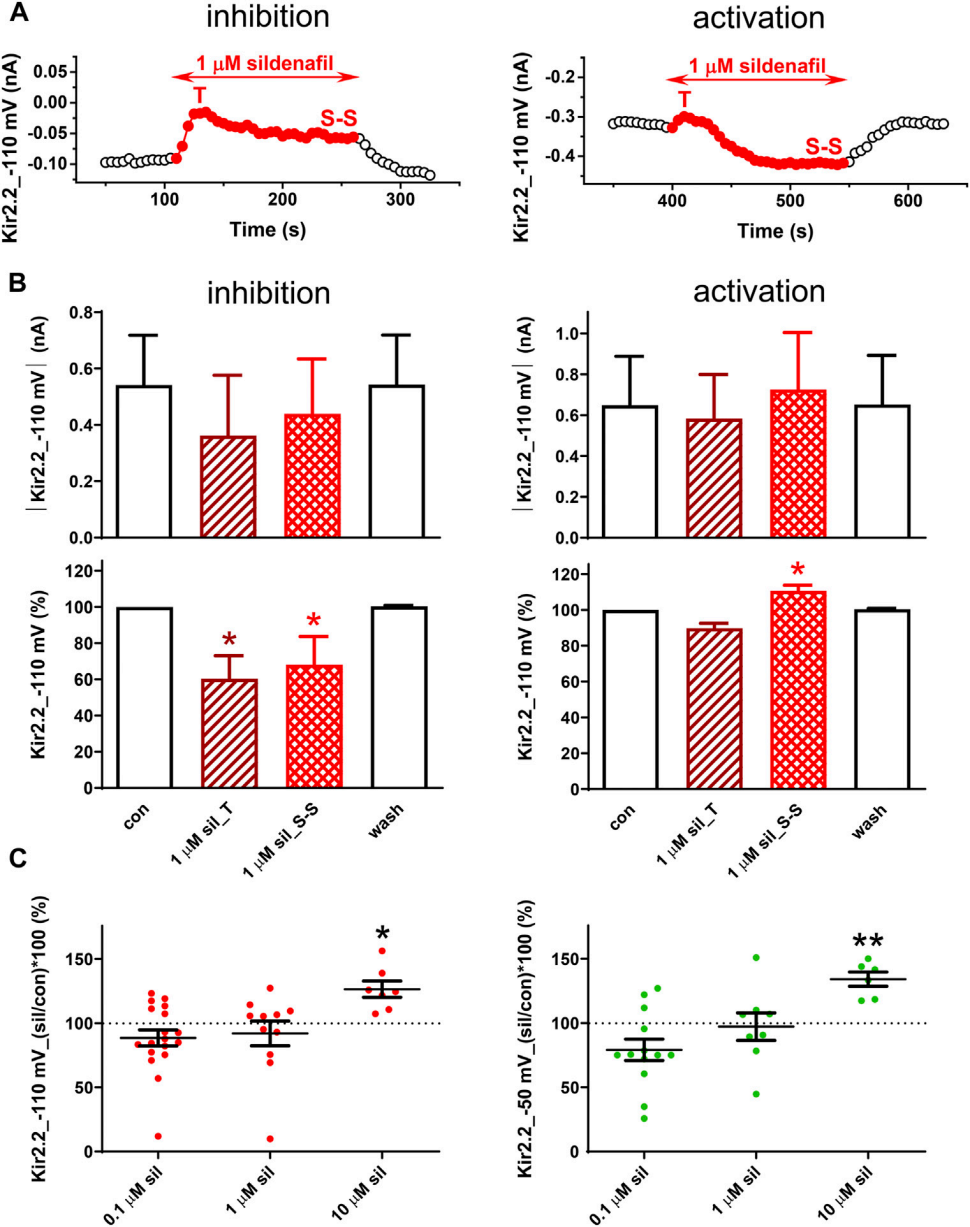
Effect of sildenafil on the human Kir2.2 channels. **(A)** Representative time courses of Kir2.2 changes in control (con), during application of 1 µM sildenafil (sil), and during the subsequent wash-out (wash); T—transient effect, S-S—steady-state effect. **(B)** Kir2.2 current and its average changes under 1 µM sildenafil (inhibition—*n* = 5; activation—*n* = 6). **(C)** Concentration dependence of the steady-state effect of sildenafil on the human Kir2.2 (*n* = 6–19); * and ** - statistical significance at *p* < 0.05 and 0.01, respectively.

In the human Kir2.2 channels, the effect of 1 µM sildenafil was dual, first resulting in a transient inhibition (T) within 22.0 ± 2.6 s and 21.0 ± 3.2 s at both −110 and −50 mV (*n* = 9 and 5, *p* > 0.05), respectively, in all tested cells (Figure 3A). Subsequently, we observed either a reduction of this inhibition reaching a steady-state inhibition (S-S in Figure 3A, left panel) or even a steady-state activation of the measured membrane current occurred (S-S in Figure 3A, right panel). The same is reflected in the average graphs, both in the absolute and relative scales, and both the steady-state inhibition and activation were statistically significant in the relative scale when evaluated separately (Figure 3B, left and right panels, respectively). Both the inhibitory and activation sildenafil effects were fully reversible during the subsequent wash-out.

Due to the dual character of the sildenafil effect on Kir2.2, the average effect was not statistically significant at 0.1 and 1 µM (Figure 3C). In contrast, a pure steady-state activation was apparent at 10 µM sildenafil and this effect was statistically significant at both −110 and −50 mV (26.4% ± 6.4% and 34.1% ± 5.6% steady-state activation at −110 and −50 mV, respectively; Figure 3C). No statistical difference was revealed in the sildenafil effect at both tested voltages.

### Impact of low Ba^2+^ concentration on the effect of sildenafil on Kir2.1 and Kir2.2 channels

**FIGURE 4:**
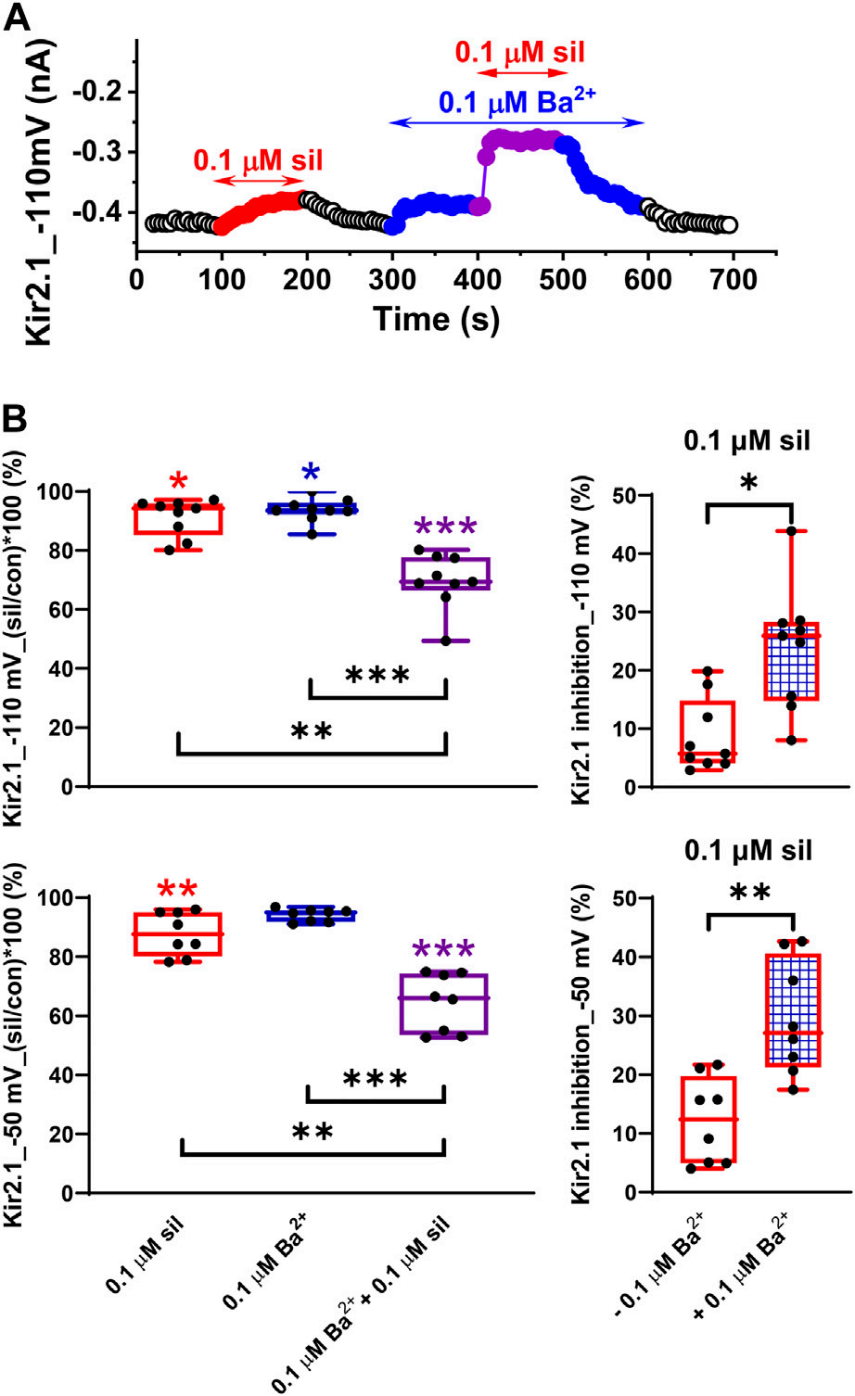
Potentiation of the effect of sildenafil on the human Kir2.1 channels by a low concentration of Ba^2+^. **(A)** Time course of Kir2.1 changes in a representative cell in control (con), during the application of 0.1 µM sildenafil (sil) alone or in combination with 0.1 µM Ba^2+^. **(B)** Effect of 0.1 µM sildenafil (sil), 0.1 µM Ba^2+^, and their combination in the relative scale at −110 and −50 mV (upper and lower graphs, respectively, in the left panel) and comparison of the effect of 0.1 µM sildenafil in the absence and presence of 0.1 µM Ba^2+^ at −110 and −50 mV (upper and lower graphs, respectively, in the right panel; *n* = 8); *, **, and ***—statistical significance at *p* < 0.05, 0.01, and 0.001, respectively (coloured stars in Figure 4B, left panel, show the statistical significance of the respective drug concentration vs. control).

Considering our recently published data on the combined effect of 0.1 µM sildenafil and 0.1 µM Ba^2+^ on *I*_K1_ in rat ventricular myocytes (Macháček et al., 2022), we decided to test this effect in the human Kir2.1 and Kir2.2 channels as well. In agreement with the data published in rats, we observed a slight inhibition of Kir2.1 in both 0.1 µM sildenafil and 0.1 µM Ba^2+^ when applied separately (8.70% ± 2.10% and 6.31% ± 1.34%, respectively, at −110 mV, and 12.2% ± 2.6% and 5.95% ± 0.75%, respectively, at −50 mV; Figures 4A, B, left panels). The combined effect was significantly higher at both tested voltages, reaching 30.3% ± 3.1% and 35.5% ± 3.4% at −110 and −50 mV, respectively. When we compared the effect of 0.1 µM sildenafil itself in both conditions, it was significantly higher in the presence of 0.1 µM Ba^2+^ (24.0% ± 3.5% and 29.5% ± 3.4% at −110 and −50 mV, respectively) than in its absence (8.70% ± 2.10% and 12.2% ± 2.6% at −110 and −50 mV, respectively; Figure 4B, right panels). No statistically significant differences between the effects at −110 and −50 mV were observed.

**FIGURE 5:**
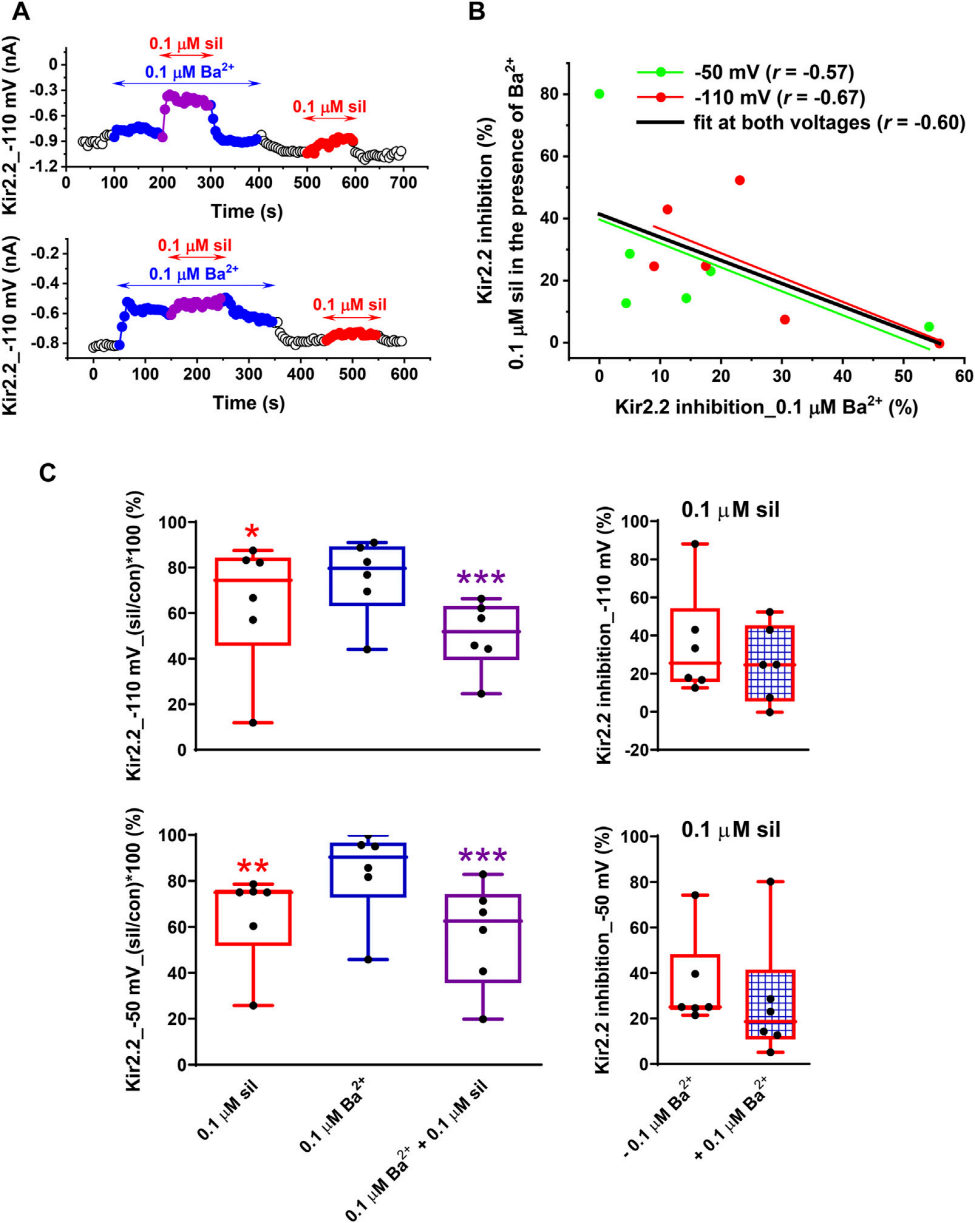
Impact of a low concentration of Ba^2+^ on the effect of sildenafil on the human Kir2.2 channels. **(A)** Kir2.2 changes at −110 mV during the application of 0.1 µM sildenafil (sil) in the absence and presence of 0.1 µM Ba^2+^ in two representative cells. **(B)** The higher was the inhibition of Kir2.2 by 0.1 µM Ba^2+^, the lower was the effect of 0.1 µM sildenafil in the presence of 0.1 µM Ba^2+^ (*i.e.,* the less likely potentiation of the sildenafil effect was apparent; if both voltages were taken into account, the Pearson´s correlation coefficient *r* = −.60, *p* < 0.05). **(C)** In contrast to Kir2.1 channels (Figure 4B, right panel), the effect of 0.1 µM sildenafil did not differ in the absence and presence of 0.1 Ba^2+^ on average, both at −110 and at −50 mV (*n* = 6); *, **, and ***—statistical significance at *p* < 0.05, 0.01, and 0.001, respectively.

In contrast to the above-described potentiation of the inhibitory effect of 0.1 µM sildenafil by 0.1 µM Ba^2+^ in Kir2.1 (Figure 4), the effect was not so clear in Kir2.2. Although a similar potentiation of the sildenafil effect could be demonstrated in some cells (Figure 5A, upper panel), no such effect was apparent in others (Figure 5A, lower panel). The final effect of 0.1 µM sildenafil in the presence of 0.1 µM Ba^2+^ seemed to be significantly dependent on the actual Kir2.2 inhibition by 0.1 µM Ba^2+^ - the lower was the inhibition by Ba^2+^, the more likely potentiation of the sildenafil effect in the presence of Ba^2+^ could be observed (for data at both −110 and −50 mV, the Pearson´s correlation coefficient *r* = −0.60, *p* < 0.05; Figure 5B; for a potential explanation, see Discussion). The average Kir2.2 data showed no potentiation of the sildenafil effect by Ba^2+^ at both −110 and −50 mV (Figure 5C, left panels), the inhibition by 0.1 µM sildenafil in the presence of 0.1 µM Ba^2+^ was even insignificantly lower (25.3% ± 8.2% and 27.3% ± 11.1% at −110 and −50 mV, respectively) than the inhibition by 0.1 µM sildenafil alone (35.2% ± 11.6% and 35.0% ± 8.3% at −110 and −50 mV, respectively; Figure 5C, right panels).

## Discussion

This is the first study showing the effect of sildenafil on the human inward rectifier potassium (Kir) channels. As observed, sildenafil at 0.1–10 µM inhibited Kir2.1 channels and the effect at 0.1 µM was significantly potentiated by a low Ba^2+^ concentration of 0.1 µM. For Kir2.2, sildenafil exhibited a dual effect at both subtherapeutic and therapeutic concentrations of 0.1 and 1 µM while demonstrating an activation at the supratherapeutic concentration of 10 µM. The potentiation by 0.1 µM Ba^2+^ tested at 0.1 µM sildenafil was apparent only in cells with low Ba^2+^-induced Kir2.2 inhibition, but no significant changes were observed on average.

Since Kir2.1 is the prevalent subunit forming the rodent ventricular *I*_K1_ channels in the working myocardium (Panama et al., 2007), it is not surprising that our data on Kir2.1 resemble those previously published on *I*_K1_ in isolated rat ventricular myocytes (Macháček et al., 2022). According to the available literature, a similar Kir2.x subunit expression pattern is present in human ventricular *I*_K1_ (Wang et al., 1998; Gaborit et al., 2007; for a recent review, see Reilly and Eckhardt 2021). Hence, we presume that the effects observed using the expressed human Kir2.1 channels in this study should be very similar to those that could be observed in human cardiomyocytes.

The effects of sildenafil on Kir currents might be caused either by direct interaction of the drug with the channels, or other ways may be responsible or contribute to the effect, for example, the secondary messenger pathways including those related to cGMP and cAMP. Since sildenafil is a well-known PDE5 inhibitor (Turko et al., 1999) and PDE5 expression as well as its activity may be detected even in healthy cardiomyocytes (Shan et al., 2012; Garcia et al., 2018), cGMP can be accumulated in cardiomyocytes during sildenafil use. Despite no data on the effect of cGMP on the cardiac Kir channels being available to our knowledge, this secondary messenger was shown to inhibit the Kir channels in endothelial cells (Shimoda et al., 2002) where these channels are preferentially represented by Kir2.1 (Sonkusare et al., 2016). Therefore, the possible role of cGMP in the observed sildenafil-induced Kir channel inhibition cannot be excluded. PDE5 activity is significantly increased in diseased myocardium, namely in failing hearts (Shan et al., 2012; Garcia et al., 2018), which might substantially increase the risk of sildenafil-induced proarrhythmic side effects in these patients if cGMP would be involved in the proarrhythmic effect of sildenafil. Despite the high sildenafil selectivity to PDE5 over PDE3 (4000-folds), PDE3 inhibition may still cause considerable changes in the tissues with predominant expression of PDE3, including the myocardium (Cheitlin et al., 1999). Moreover, cGMP is known to inhibit PDE3 (Degerman et al., 1997) which implies that it might directly elevate the cAMP level in cardiomyocytes. Therefore, increased cAMP levels and activation of protein kinase A (PKA), which are known to affect the Kir channels and decrease their function (Koumi et al., 1995a; b), might also play a role in the sildenafil effect on these channels. Further investigation is needed to reveal the molecular mechanisms of sildenafil-Kir channel interaction.

The potentiation of the sildenafil inhibitory effect on Kir2.2 was observed only in the cells with low reactivity to Ba^2+^-induced inhibition (Figures 5A, B). On average, these changes were not significant because the inhibitory effect of Ba^2+^ on Kir2.2 was too high, preventing the potentiation as has been also observed at higher Ba^2+^ inhibition in our recent study on rat *I*_K1_ (Macháček et al., 2022). The inhibition of Kir2.2 induced by Ba^2+^ was higher than that of Kir2.1 (at −110 mV: 24.5% ± 7.0% in Kir2.2 vs. 6.31% ± 1.34% in Kir2.1, *n* = 6, *p* < 0.01; at −50 mV: 16.0% ± 8.1% in Kir2.2 vs. 5.95% ± 0.75% in Kir2.1, *n* = 8, *p* > 0.05). This observation agrees with previously published studies which also confirmed higher sensitivity of Kir2.2 channels to Ba^2+^ in comparison to Kir2.1 (Schram et al., 2003; Panama et al., 2010).

No significant differences were observed between the effect of sildenafil and/or Ba^2+^ on the inward and outward Kir current components (*i.e.,* at −110 and −50 mV, respectively). Hence, the data are physiologically relevant and the impact of the revealed Kir changes on action potential configuration is likely. It well agrees with our previously published data on rat ventricular *I*_K1_ showing a significant action potential prolongation during the combined application of sildenafil and Ba^2+^ (Macháček et al., 2022). Moreover, both sildenafil and Ba^2+^ were applied in concentrations that can be identified in the human body. The peak plasma concentration of sildenafil for a therapeutic dose of oral sildenafil (25–100 mg) was reported to vary between 127 and 560 μg/L (268–1180 nM; Nichols et al., 2002). Ba^2+^ is an environmental contaminant with plasma concentrations between 1 and 60 μg/L (7–437 nM) in a common population (Łukasik-Głębocka et al., 2014). Our previous observation of the impact of potentiated inhibition in the presence of 0.1 µM sildenafil and 0.1 µM Ba^2+^ on ventricular cell repolarization (Macháček et al., 2022) suggests that a transient increase in the plasma concentration of environmental contaminant Ba^2+^ could contribute to the genesis of arrhythmias in patients treated with sildenafil.

Arrhythmogenesis related to the use of sildenafil is likely complex. Besides the effect of sildenafil on the Kir channels demonstrated in this study as well as in our previous study (Macháček et al., 2022), changes of other cardiac ionic currents should be considered. A study on the rapid component of delayed rectifier potassium current (*I*_Kr_) demonstrated that sildenafil exerted a reversible inhibitory effect on *I*_Kr_ channels expressed in a cell line, but the inhibition was below 10% at the therapeutic sildenafil concentration of 1 µM (the half inhibitory concentration of ∼100 µM); the supratherapeutic concentration (30 µM) inhibiting ∼44% of *I*_Kr_ in the cell line showed a prolongation cardiac repolarization by 15% in isolated guinea pig heart (Geelen et al., 2000). Surprisingly, an opposite effect on the action potential duration, *i.e.* its shortening, was observed at supratherapeutic sildenafil concentrations above 10 µM in guinea pig papillary muscles and canine Purkinje fibers by Chiang et al. (2002). This study performed on guinea pig ventricular myocytes also did not confirm the inhibitory effect of sildenafil on *I*_Kr_ described before by Geelen et al. (2000), even at 30 µM sildenafil. Therefore, the effect of sildenafil on *I*_Kr_ does not seem to considerably contribute to changes of cardiac cell electrophysiology in the presence of sildenafil, especially at its therapeutic concentrations. No effect on the slow component of delayed rectifier potassium current (*I*_Ks_) and on persistent sodium current (*I*_pNa_) was also apparent in guinea pig ventricular myocytes, even at 30 µM sildenafil (Chiang et al., 2002). In contrast, Chiang et al., 2002 demonstrated that sildenafil significantly inhibited L-type calcium current (*I*_Ca,L_) in guinea pig ventricular myocytes, which explained the above-mentioned action potential shortening observed by this research group at supratherapeutic sildenafil concentrations above 10 μM; however, only a slight inhibition was apparent in the presence of 1 µM sildenafil (the half inhibitory concentration was 27.2 µM). Considering all these facts, a significant, but slight contribution to the clinically relevant sildenafil effects on cardiac cell electrophysiology may be expected only by *I*_Ca,L_ (Chiang et al., 2002) and *I*_K1_ (Macháček et al., 2022 and this study). The concurrent slight inhibitions of the depolarizing *I*_Ca_ and the repolarizing *I*_K1_ at therapeutic concentrations of sildenafil, which induce an opposite impact on action potential repolarization, may explain the previously reported absence of significant changes of action potential duration and QT interval under therapeutic sildenafil doses (*e.g.,* Sugiyama et al., 2001; Chiang et al., 2002; Alpaslan et al., 2003; Kaya et al., 2004). In contrast, during the combined application of 0.1 µM sildenafil and 0.1 µM Ba^2+^, we observed a high inhibition of *I*_K1_ and Kir2.1 (*I*_K1_: ∼46% at −50 mV, Figs. 3 and 4 in Macháček et al., 2022; Kir2.1: ∼36% at −50 mV, Figure 4 in this study) and significant prolongation of rat ventricular action potential (by ∼20% - Fig. 5 in Macháček et al., 2022). It implies that the Kir current changes may prevail under these conditions and may exert a proarrhythmic effect in some patients.

Besides the effect of sildenafil on cardiac ionic currents, the ability of this drug to cause vasodilation and consequent reflex sympathetic stimulation preventing the drop of systemic blood pressure (*e.g.,* Phillips et al., 2000) should be also taken into account when the arrhythmogenic mechanisms of sildenafil are considered. The proarrhythmic potential of sympathetic stimulation is well known (besides others, it also suppresses *I*_K1_ - Koumi et al., 1995a; b). A potential proarrhythmic factor related to *I*_K1_ inhibition induced by sildenafil might be also vasoconstriction due to a decreased repolarization force in vascular smooth muscles. Nevertheless, the opposite vasodilatory effect and increased coronary flow were apparent in healthy coronary arteries (Ishikura et al., 2000). This is not surprising considering the basic sildenafil inhibitory effect on PDE5 and the ability of cGMP to open the Ca^2+^-activated potassium channels (Robertson et al., 1993) as well as another subtype of the Kir channels, the ATP-sensitive Kir (*I*_K(ATP)_) channels, in vascular smooth muscle cells (Kubo et al., 1994). The similar effect of cGMP on the cardiac *I*_K(ATP)_ channels (Chai et al., 2011) and especially the ability of sildenafil to open the mitochondrial *I*_K(ATP)_ channels (Salloum et al., 2007) may at least partly explain the origin of the cardioprotective effect of sildenafil against ischemia-reperfusion injury (Salloum et al., 2007). In contrast, a decreased flow was described in coronary arteries with critical stenosis if sildenafil was used in combination with nitrates (Ishikura et al., 2000). This undesired effect resulted from excessive vasodilation caused by this drug combination leading to a large drop of systemic blood pressure. In any case, most of the reported cardiovascular deaths associated with the use of sildenafil did not happen in patients treated with nitrates (*e.g.,* Azarbal et al., 2000).

We conclude that sildenafil significantly affects the human Kir channels, showing namely a pure inhibition of the Kir2.1 channels. This inhibition was significantly potentiated when sildenafil at a subtherapeutic concentration of 0.1 µM was applied concurrently with the same low Ba^2+^ concentration which might be commonly reached in the human body due to environmental contamination. These effects are in line with the effect of sildenafil in rat ventricular *I*_K1_ which was shown to significantly delay ventricular repolarization in rat ventricular myocytes. With respect to the similar expression patterns in the rodent and human ventricular *I*_K1_ channels, analogical sildenafil effects on *I*_K1_ may be expected in human ventricular myocytes. Considering the published data on the electrophysiological effects of sildenafil as well as our new findings on its effects on *I*_K1_ channels, the risk of the development of arrhythmia seems to be generally low if no risk factor can be identified in the patient and the dosing is standard. This study identifies the presence of Ba^2+^ in the human body as a new risk factor for arrhythmia occurrence in patients treated with sildenafil.

## Data Availability

The raw data supporting the conclusion of this article will be made available by the authors, without undue reservation.
